# Comparative study of acid- and alkali-catalyzed 1,4-butanediol pretreatment for co-production of fermentable sugars and value-added lignin compounds

**DOI:** 10.1186/s13068-023-02303-5

**Published:** 2023-03-28

**Authors:** Xinyu Xie, Mingjun Chen, Wenyao Tong, Kai Song, Jing Wang, Shufang Wu, Jinguang Hu, Yongcan Jin, Qiulu Chu

**Affiliations:** 1 Jiangsu Co-Innovation Center of Efficient Processing and Utilization of Forest Resources, International Innovation Center for Forest Chemicals and Materials, College of Light Industry and Food Engineering, Nanjing Forestry University, Nanjing, 210037 China; 2 College of Biology and the Environment, Nanjing Forestry University, Nanjing, 210037 China; 3 Guangdong Provincial Key Laboratory of Silviculture, Protection and Utilization, Guangdong Academy of Forestry, Guangzhou, 510520 China; 4 Department of Chemical and Petroleum Engineering, University of Calgary, 2500 University Dr. NW, Calgary, AB T2N 1Z4 Canada

**Keywords:** Organosolv pretreatment, Enzymatic hydrolysis, BDO lignin, Antioxidant activity

## Abstract

**Background:**

Organosolv pretreatment is one of the most efficient methods for delignification and boosting biomass saccharification. As compared to typical ethanol organosolv pretreatments, 1,4-butanediol (BDO) organosolv pretreatment is a high-boiling-point solvent pretreatment, which can generate low pressure in the reactor during high temperature cooking that improves the operation safety. Although several studies showed that organosolv pretreatment can lead to effective delignification and enhancement in glucan hydrolysis, there has been no studies on acid- and alkali-catalyzed BDO pretreatment, as well as their comparison on promoting biomass saccharification and lignin utilization.

**Results:**

It was shown that BDO organosolv pretreatment was more effective in removing lignin from poplar as compared with typical ethanol organosolv pretreatment under the same pretreatment conditions. HCl-BDO pretreatment with 40 mM acid loading led to 82.04% of original lignin removed from biomass, as compared to the lignin removal of 59.66% in HCl-Ethanol pretreatment. Besides, acid-catalyzed BDO pretreatment was more effective in improving the enzymatic digestibility of poplar than alkali-catalyzed BDO pretreatment. As a result, HCl-BDO with acid loading of 40 mM provided a good enzymatic digestibility of cellulose (91.16%) and the maximum sugar yield of 79.41% from original woody biomass. The linear correlations between physicochemical structure (e.g., fiber swelling, cellulose crystallinity, crystallite size, surface lignin coverage and cellulose accessibility) changes of BDO pretreated poplar and enzymatic hydrolysis were plotted to figure out the main factors that influenced biomass saccharification. Moreover, acid-catalyzed BDO pretreatment mainly brought about the phenolic hydroxyl (PhOH) groups formation in lignin structure, while alkali-catalyzed BDO pretreatment mostly led to the lower molecular weight of lignin.

**Conclusions:**

Results indicated that the acid-catalyzed BDO organosolv pretreatment could significantly improve enzymatic digestibility of the highly recalcitrant woody biomass. The great enzymatic hydrolysis of glucan resulted from increased cellulose accessibility, which mostly associated with the higher degree of delignification and hemicellulose solubilization, as well as the more increase in fiber swelling. Besides, lignin was recovered from the organic solvent, which could be used as natural antioxidants. The formation of phenolic hydroxyl groups in lignin structure and the lower molecular weight of lignin contributed to its greater radical scavenging capacity.

## Background

Lignocellulosic biomass represents the abundant and renewable feedstock for the production of bio-based chemicals and fuels under the concept of sustainable biorefinery [1]. Poplar is proposed as a very promising biomass for biorefinery, owning to its relatively high content of carbohydrate and rapid growth rate. The bio-based ethanol production process from lignocellulosic biomass like poplar comprises pretreatment, enzymatic hydrolysis, fermentation and distillation, in which the pretreatment plays a key role in improving the ease of subsequent enzymatic hydrolysis and promoting the overall biomass utilization [2, 3].

Different pretreatment techniques, including physical, chemical and biological methods, have been applied to biomass, individually or in combination, to increase the yield and productivity of the desired products [4, 5]. Among chemical pretreatments, acid pretreatment is identified as one of the most promising pretreatment methods due to its easy operation, low cost and efficient processing of a large range of biomass [6]. During acid pretreatment, hemicellulose is effectively solubilized, resulting in improved ease of glucan hydrolysis to some extent [7]. However, there is still a considerable amount of lignin that remains in the pretreated substrates, which restricts subsequent enzymatic hydrolysis [8]. Moreover, lignin undergoes repolymerization reaction, which impairs glucan hydrolysis and hinders downstream utilization of lignin [9]. Thereby, it is essential to modify the acid pretreatment to simultaneously remove hemicellulose and lignin to promote biomass saccharification, while allowing possible valorization of recovered lignin.

It had been proposed by previous work that, the acid-catalyzed organosolv pretreatment, like acid–ethanol pretreatment, can effectively remove hemicellulose and lignin together [10], resulting in the cellulose-enriched solid with improved ease of glucan hydrolysis [6]. Other organosolv pretreatments such as 1,4-butanediol (BDO), gamma-valerolactone (GVL) also have been successfully applied to pretreat biomass for the fractionation of high-quality lignin and cellulose, while increasing the biomass saccharification [11, 12]. As compared to typical ethanol organosolv pretreatments, a more significant amount of lignin is anticipated to be removed after organosolv pretreatment using 1,4-butanediol (BDO), likely due to the greater solvent solubility [12]. In addition, it is proposed that 1,4-butanediol (BDO) pretreatment suppresses lignin repolymerization, which has potential to reduce lignin inhibition on glucan hydrolysis [13] and to promote downstream utilization of lignin [14]. Moreover, BDO is a high-boiling-point solvent with a boiling temperature of 232 °C, which can generate low pressure in the reactor during high temperature cooking that improves the operation safety [15]. Like γ-valerolactone, the liquid stream after pretreatment could be enriched in extracted C5 sugars and lignin, which had the potential to be converted into various value-added chemicals [11]. For example, the precipitated lignin benefited from high phenolic content and reduced molecular weight, which could be utilized as antioxidants [16], and the dissolved hemicellulose could be upgraded to furanic platform chemicals [17]. Moreover, it had been demonstrated that BDO could be well recovered from water using Mitsubishi SP70 adsorbent, a kind of macroporous adsorption resin, instead of energy-consuming vaporization process for BDO recovery [18]. After separation through adsorption, BDO could be reused in the pretreatment of lignocellulosic biomass [19].

Among various acids, sulfuric acid is the usual acid employed, however, it has been reported that hydrochloric acid is superior to sulfuric acid in hemicellulose depolymerization [20, 21], which is supposed to further increase cellulose accessibility to cellulase enzymes. Compared with acidic-catalyzed alcohol pretreatment, alkali-catalyzed alcohol pretreatment is probably more effectively in removing lignin from biomass [6]. It is shown by previous work that there is a synergistic effect of alcohol and alkali on biomass delignification, and the pretreatment severity can be significantly reduced through the addition of NaOH and alcohol [22]. Furthermore, the combination of high-boiling-point alcohol, like ethylene glycol, and alkali can reserve more cellulose and hemicellulose while producing less inhibitors [6], in addition to a greater degree of delignification, which may be beneficial for subsequent enzymatic hydrolysis and fermentation.

Although several studies showed that organosolv (like ethanol, ethylene glycol) pretreatment can lead to effective delignification and enhancement in glucan hydrolysis [6, 10], there has been no studies on acid- and alkali-catalyzed BDO pretreatment, as well as their comparison, which may have the potential to promote the biomass saccharification and lignin utilization. Thus, this work initially focused on the contrastive analysis of various acid- and alkali-catalyzed BDO pretreatments, with a comprehensive study on the influence of various BDO pretreatments on chemical compositions, physiochemical properties, cellulose accessibility and hydrolysis efficiency of biomass. The linear correlations between physicochemical structure (*e.g.*, fiber swelling, cellulose crystallinity, crystallite size, surface lignin coverage and cellulose accessibility) changes of BDO pretreated poplar and enzymatic hydrolysis were plotted to figure out the crucial factors in impacting biomass saccharification. Moreover, lignin is a biodegradable, abundant and ecofriendly biopolymer, making it a suitable antioxidant for use in polymeric materials [16]. Therefore, the effect of modified BDO pretreatments on the yield, physiochemical properties, and antioxidant activity of recovered lignin was also studied in this work. Finally, the mass balance analysis based on modified BDO pretreatment was proposed for co-production of glucose, xylose, and antioxidant lignin from highly recalcitrant woody biomass for holistic utilization of lignocellulosic biomass under sustainable biorefinery concept.

## Results and discussion

### Effect of organosolv pretreatment on chemical composition of biomass

Organosolv pretreatment has been shown to be a promising strategy for lignin removal from lignocellulosic biomass [13]. Thus, organosolv pretreatment using ethanol and BDO was performed on poplar sawdust, respectively. The chemical compositions of raw biomass and pretreated substrates are shown in Table 1. It was observed that lignin was selectively removed in acid-catalyzed ethanol organosolv pretreatment. With increase in acid loading from 30 to 50 mM, lignin removal enhanced from 39.30% to 66.51%, contributing to greater cellulose accessibility from 134.76 to 197.58 mg/g (Table 2). However, carbohydrate, in particular hemicellulose, was also solubilized to a greater extent, evidenced by the lower hemicellulose retention in WIF (Table 2). This was because a larger amount of acid helped disrupt the ester bonds linking lignin and hemicellulose, while cleaving glycosidic bonds of hemicellulose polysaccharide [6], resulting in lower hemicellulose recovery in WIF ranging from 62.89% to 27.23% (Table 2). In this scenario, delignification selectivity was used to evaluate the efficacy of organosolv pretreatment, which was defined as gram lignin removed from biomass per gram carbohydrate (cellulose and hemicellulose) removed. As acid loading increased from 30 to 50 mM in ethanol organosolv pretreatment, it was shown that delignification selectivity reduced from 1.10 to 0.95 due to the more significant carbohydrate solubilization than lignin removal during pretreatment (Table 2).

**Table 1:** Chemical compositions of untreated and pretreated substrates

Pretreatment		Cellulose (%)	Hemicellulose (%)	Lignin (%)
Raw biomass		46.38	20.50	29.03
HCl-Ethanol	30 mM	58.17	16.73	22.87
HCl-Ethanol	40 mM	67.40	11.60	18.32
HCl-Ethanol	50 mM	72.47	9.63	16.78
BDO	No catalyst	52.21	20.46	25.74
HCl-BDO	10 mM	56.78	20.48	20.75
HCl-BDO	20 mM	66.69	14.97	15.58
HCl-BDO	30 mM	73.06	11.27	13.10
HCl-BDO	40 mM	78.60	11.42	9.57
HCl-BDO	50 mM	88.60	5.00	6.14
NaOH-BDO	250 mM	61.81	16.59	19.94
NaOH-BDO	300 mM	65.07	15.63	17.37
NaOH-BDO	350 mM	67.60	14.58	16.10
NaOH-BDO	400 mM	70.70	13.12	13.97
NaOH-BDO	450 mM	72.84	11.44	13.87

**Table 2:** Effect of organosolv pretreatment on carbohydrate retention and lignin removal

	Cellulose retention in WIF (%)	Hemicellulose retention in WIF (%)	Hemicellulose removal (%)	Lignin removal (%)	Delignification selectivity	Cellulose accessibility (mg/g)
HCl-Ethanol 30 mM	96.63	62.89	37.11	39.30	1.10	134.76
HCl-Ethanol 40 mM	92.90	36.17	63.83	59.66	1.01	181.33
HCl-Ethanol 50 mM	90.54	27.23	72.77	66.51	0.95	197.58
BDO-No catalyst	98.77	87.57	12.43	22.20	2.07	92.39
HCl-BDO 10 mM	99.43	81.15	18.85	41.95	2.95	103.01
HCl-BDO 20 mM	96.63	49.06	50.94	63.92	1.55	198.41
HCl-BDO 30 mM	96.58	33.69	66.31	72.34	1.39	256.47
HCl-BDO 40 mM	92.30	30.35	69.65	82.04	1.34	305.51
HCl-BDO 50 mM	90.25	11.51	88.49	90.00	1.15	326.16
NaOH-BDO 250 mM	96.65	58.68	41.32	50.20	1.54	173.27
NaOH-BDO 300 mM	96.16	52.28	47.72	58.99	1.48	186.84
NaOH-BDO 350 mM	95.35	46.54	53.46	63.72	1.41	201.46
NaOH-BDO 400 mM	93.14	39.09	60.91	70.60	1.31	223.76
NaOH-BDO 450 mM	89.79	31.91	68.09	72.69	1.13	247.90

Cleary, BDO pretreatment was more effective in removing lignin from poplar at the same acid loading (30–50 mM, Table 1). As shown, HCl-BDO pretreatment at 40 mM acid led to lignin removal of 82.04%, as compared to the lignin removal of 59.66% in HCl-Ethanol pretreatment, which gave rise to higher delignification selectivity of 1.34 (Table 2). It was suggested that BDO had a smaller relative energy difference (RED) with lignin than other alcohols like ethanol [13], bringing about a better efficiency for lignin extraction [6].

Compared with acid-catalyzed organosolv pretreatment, alkali-catalyzed organosolv pretreatment had been reported to reserve more cellulose and hemicellulose, while producing less inhibitors towards enzymatic hydrolysis [22]. As suggested, the higher hemicellulose retention in pretreated solids was favorable to obtain higher total fermentable sugars concentration after enzymatic hydrolysis, which improved the end-product tier after fermentation [23]. When NaOH-BDO pretreatment was performed on poplar sawdust, it was worth noting that much higher NaOH loading (250–450 mM) was applied as compared to acid BDO pretreatment (10–50 mM). Besides, at similar delignification selectivity around 1.3 (NaOH 400 mM), more hemicellulose was reserved in water-insoluble fraction (WIF) after NaOH-BDO pretreatment, with comparison to HCl-BDO pretreatment (39.06% vs. 30.35%, Table 2). However, less lignin was removed (70.60% vs. 82.04%, Table 2), leading to the reduced cellulose accessibility as indicated by the lower DR28 adsorption to biomass (223.76 vs. 305.51 mg/g, Table 2). Results indicated the greater efficacy of acid-catalyzed BDO pretreatment on delignification in comparison with alkali-catalyzed BDO pretreatment, which promoted cellulose accessibility and might favor subsequent enzymatic hydrolysis of glucan.

As reported, acid disrupted the ester bonds linking lignin and hemicellulose, while cleaving glycosidic bonds of hemicellulose polysaccharide [24]. Thus, acid addition facilitated hemicellulose removal. Meanwhile, BDO, the organic solvent, favored the removal of lignin components [13]. Probably, the synergistic effect of HCl and BDO rendered the HCl-BDO pretreatment more efficient for both hemicellulose (~ 88.49%) and lignin (~ 90.00%) remove, as elucidated by previous work that looked at HCl-catalyzed ethylene glycol organosolv pretreatment [6]. On the other hand, acid-catalyzed BDO pretreatment had a greater efficacy in delignification in comparison with alkali-catalyzed BDO pretreatment, likely because partial alkali was consumed by the acid released from hemicellulose components, in particular the acetic acid formed by the release of acetyl groups [25].

### Effect of organosolv pretreatment on biomass saccharification

**Fig. 1:**
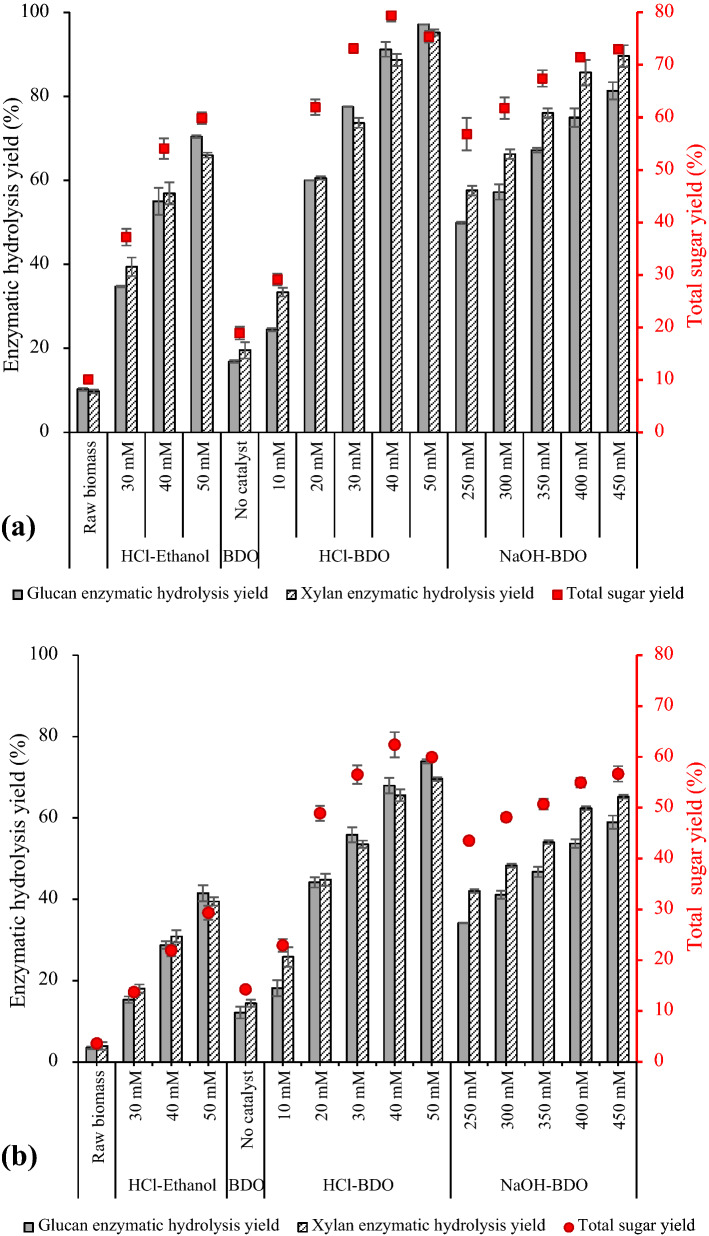
Enzymatic hydrolysis of glucan and xylan, as well as total sugar yield from biomass (**a**: 20 FPU/g; **b**: 10 FPU/g)

To figure out the effect of organosolv pretreatment on enzymatic digestibility of poplar biomass, enzymatic hydrolysis was carried out on the untreated and organosolv pretreated biomass (Fig. 1). As shown, around 10% of the glucan and xylan in untreated poplar could be enzymatically hydrolyzed at high cellulase loading of 20 FPU/g (Fig. 1a), indicating the recalcitrance of raw biomass towards enzymatic degradation, likely due to the compact lignocellulosic structure. After HCl-Ethanol organosolv pretreatment (30–50 mM), glucan hydrolysis yield was obviously enhanced, ranging from 34.76% to 70.41%. Besides, glucan hydrolysis yield was further increased to 77.47–97.08% after HCl-BDO pretreatment at the same acid loading of 30–50 mM, mainly ascribed to the greater degree of delignification and hemicellulose solubilization, which caused significantly higher cellulose accessibility for cellulase’s attack (Table 2). At lower cellulase loading of 10 FPU/g, glucan hydrolysis yield was much lower (Fig. 1b).

To directly evaluate the efficacy of pretreatment process in recovering carbohydrates while improving ease of enzymatic hydrolysis, the concept of total sugar yield from biomass was proposed, which was the sum of the sugars present in the hemicellulose-enriched WSF after pretreatment and the sugars released during enzymatic hydrolysis of the pretreated WIF (Fig. 1). It was clear that sugar yield increased with higher acid loading in BDO pretreatment. When acid loading raised from 10 to 40 mM, total sugar yield increased from 29.43% to 79.41%, then decreased to 75.35% with acid loading further increased to 50 mM (Fig. 1). This phenomenon was mainly because higher pretreatment acidity caused greater sugar degradation, the better hydrolysability of WIF after pretreatments did not offset the sugar loss during pretreatment using 50 mM acid, resulting in a lower sugars yield. Thus, the maximum sugar yield of 79.41% from original biomass was achieved after HCl-BDO pretreatment  with 40 mM HCl loading (Fig. 1).

**Fig. 2:**
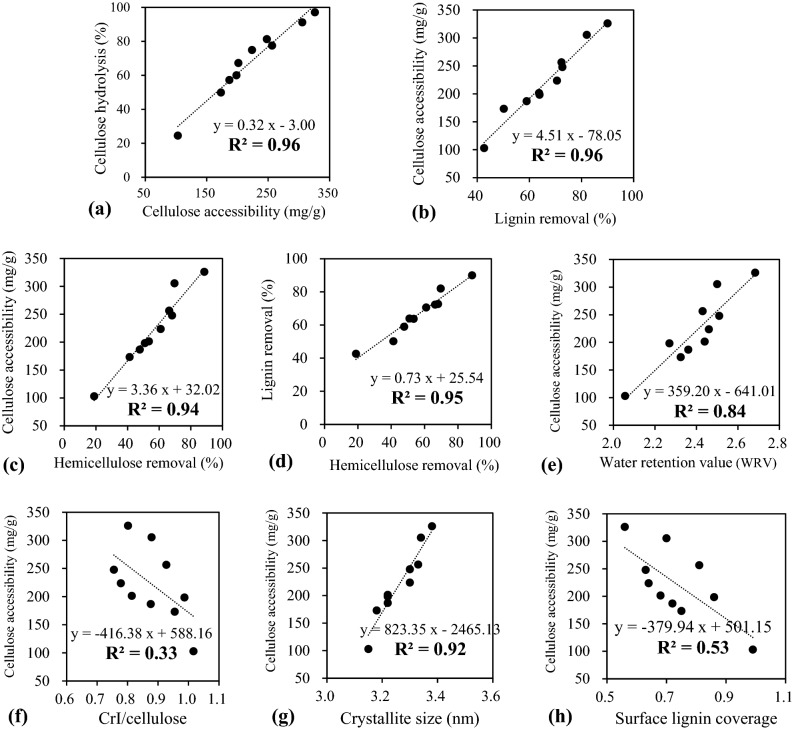
Relationship between enzymatic digestibility and cellulose accessibility vs. physiochemical properties of BDO pretreated biomass

As for NaOH-BDO pretreatment, it was also observed that the glucan hydrolysis yield improved with higher alkali loading (Fig. 1). When NaOH loading increased from 250 to 400 mM, glucan hydrolysis yield improved from 49.88% to 74.93%, while sugar yield ranged from 56.84% to 71.46%. However, with further increased NaOH loading to 450 mM, marginal increase in the sugar yield was gained (Fig. 1a). Thus, for NaOH-BDO pretreatment, NaOH loading of 400 mM was likely sufficient to obtain reasonable enzymatic hydrolysis and sugar yield from poplar biomass. Besides, it was worth noting that the enzymatic hydrolysis of glucan in NaOH-BDO pretreated biomass apparently poorer than that in HCl-BDO pretreated biomass, probably because of the lower cellulose accessibility (Fig. 2a), which mainly resulted from the lower delignification degree (Fig. 2b). Moreover, the lower hemicellulose removal from biomass after NaOH-BDO pretreatment (Table 2) was probably also responsible for the lower cellulose accessibility and the less susceptibility of biomass to enzymatic hydrolysis (Fig. 2c). It had been reported that hemicellulose acts as a physical barrier that limits the cellulose accessibility by occupying the outer surface of cellulose fibers and diffusing into the inter-fibrillar space through fiber pores [26]. Moreover, it was also suggested that hemicellulose solubilization and lignin removal from biomass was not separable, as evidenced by the good correlation between lignin removal and hemicellulose solubilization (Fig. 2d). This was likely because hemicellulose also affected lignin removal from biomass. Thus, hemicellulose also plays an important role in effecting cellulose accessibility [26–28].

### Relationship between enzymatic hydrolysis and physiochemical properties of biomass

It was considered the proper cellulose accessibility that resulted from hemicellulose solubilization and lignin removal played a primary role in affecting the enzymatic digestibility of biomass [29]. Other than lignin removal and hemicellulose solubilization, the physiochemical properties of substrate were also considered to play an important role. It was shown that, with increase of acid or alkali loading, the extent of fiber swelling was evidently increased, as indicated by the higher water retention value (Table 3). This was mainly because BDO pretreatment with higher acid or alkali loading promoted delignification, which reduced the hydrophobic lignin’s restriction on fiber swelling. The increased extent of fiber swelling was suggested to contribute to the greater cellulose accessibility (Fig. 2e), leading to improved ease of glucan hydrolysis.

**Table 3:** Effect of acid and alkali BDO pretreatment on substrate characteristics

Pretreatment	Water retention value (WRV)	XRD results			XPS results			
		CrI	CrI/cellulose	Crystallite size (nm)	Surface lignin coverage	C1 (%)	C2 (%)	C3 (%)
HCl-BDO 10 mM	2.06	0.58	1.02	3.15	0.99	75.55	20.33	4.12
HCl-BDO 20 mM	2.27	0.66	0.99	3.22	0.86	71.42	23.70	4.88
HCl-BDO 30 mM	2.43	0.68	0.93	3.33	0.81	61.04	34.24	4.72
HCl-BDO 40 mM	2.50	0.69	0.88	3.34	0.70	51.20	44.49	4.30
HCl-BDO 50 mM	2.68	0.71	0.80	3.38	0.56	47.91	48.23	3.86
NaOH-BDO 250 mM	2.32	0.59	0.95	3.18	0.75	57.88	40.66	1.45
NaOH-BDO 300 mM	2.36	0.57	0.88	3.22	0.72	54.90	42.95	2.16
NaOH-BDO 350 mM	2.44	0.55	0.81	3.22	0.68	49.86	47.40	2.75
NaOH-BDO 400 mM	2.46	0.55	0.78	3.30	0.64	47.94	48.46	3.59
NaOH-BDO 450 mM	2.51	0.55	0.76	3.30	0.63	45.37	48.34	6.29

**Fig. 3:**
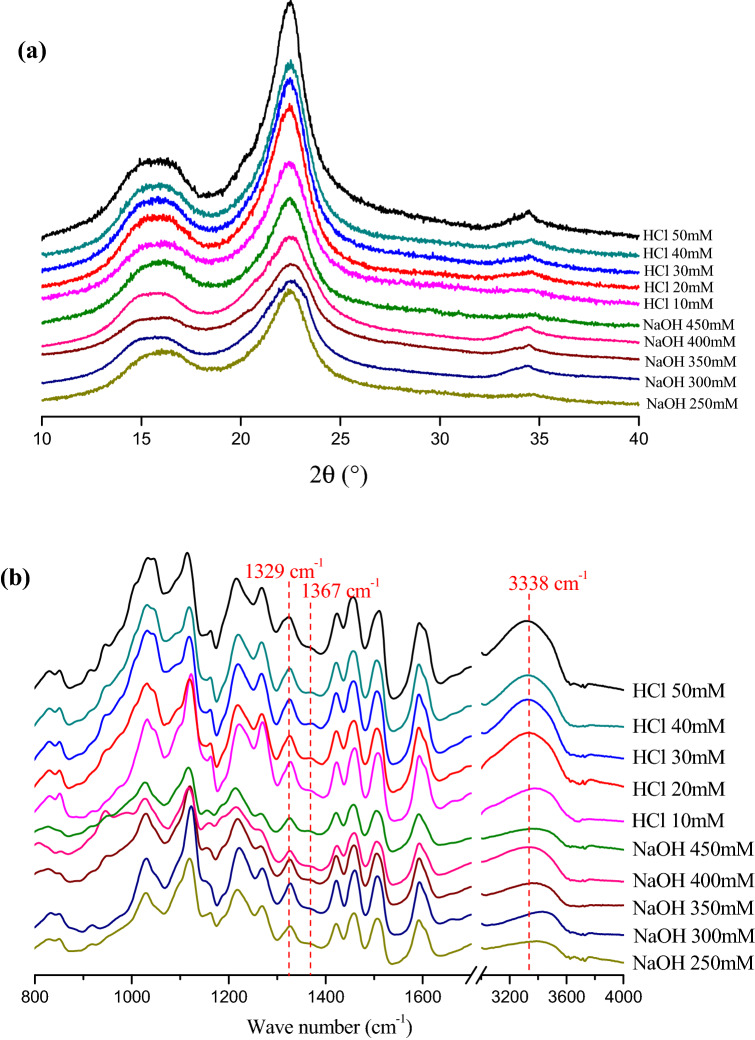
XRD spectra of BDO pretreated solid (**a**) and FTIR of recovered lignin (**b**)

Previous work showed that the cellulose crystallinity also had influence on glucan hydrolysis of lignocellulosic biomass [30]. Thus, the X-ray diffraction (XRD) analysis of diverse BDO pretreated substrates was performed (Fig. 3a). It was found that increased acid or alkali loading in BDO pretreatment caused higher crystallinity (CrI) (Table 3), as the content of cellulose in pretreated biomass was increased by higher chemical loading through greater degree of delignification and hemicellulose solubilization [6]. However, the ratio of crystallinity to cellulose (CrI/cellulose) decreased (Table 3), implying that higher catalyst loading facilitated the decrystallization effect of BDO pretreatment. Moreover, lower CrI/cellulose was detected in NaOH-BDO pretreatment at similar delignification selectivity (around 1.3) as compared to HCl-BDO (Table 3), giving a hint about the more effective decrystallization of alkaline treatment than acidic treatment, which agreed with earlier work [31]. When CrI/cellulose values of BDO pretreated biomass were fitted with their cellulose accessibility (Fig. 2f), a poor correlation was found. Result indicated that the decrystallization effect of BDO pretreatment probably favored the ease of glucan hydrolysis to some extent, but showed a marginal effect on the enzymatic digestibility of biomass.

In addition, it was also noticed BDO organosolv pretreatment that generated the highest glucose yield was accompanied with the most significant increase in crystallite size (Table 3). A good correlation was also found between cellulose accessibility and crystallite size (Fig. 2g). This was probably because BDO pretreatment boosted delignification, which alleviated the lignin’s restriction on fiber swelling and induced the swelling of cellulose [32]. As a result, swelling cellulose microfibrils could increase cellulose surface area and improve accessibility to cellulase for enzymatic hydrolysis [33]. Moreover, it was also expected that acidic treatments had a greater influence on cellulose microfibril structure [34], displaying a larger increase in the crystallite size than alkaline BDO pretreatment (Table 3).

**Fig. 4:**
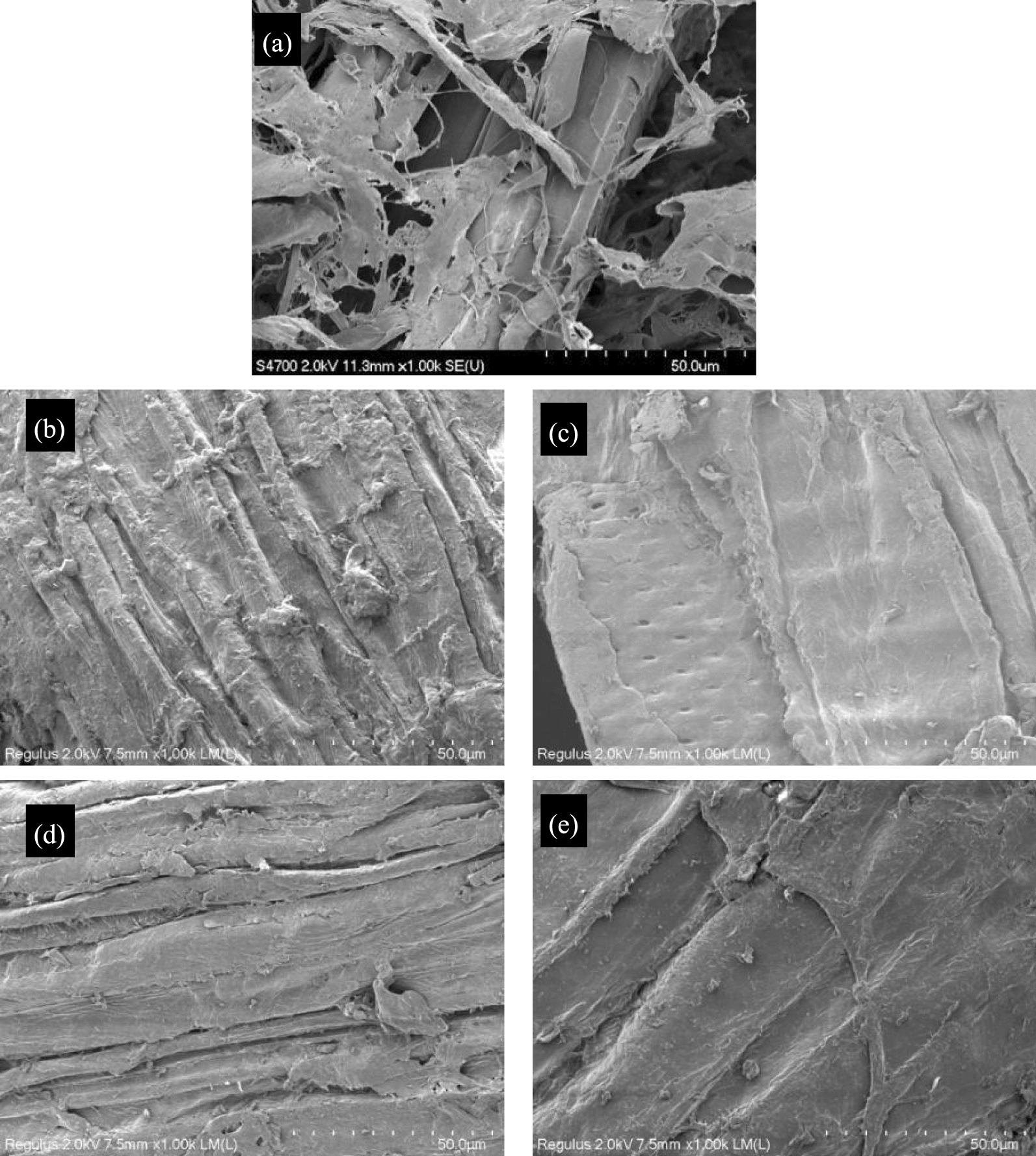
SEM observations of the raw and pretreated biomass: raw biomass (**a**); HCl-BDO pretreated solid at 10 mM (**b**) and 40 mM (**c**); NaOH-BDO pretreated solid at 250 mM (**d**) and 400 mM (**e**)

Scanning electron microscope (SEM) and X-ray photoelectron spectroscopy (XPS) were used to estimate the surface morphology changes in BDO organosolv pretreatment (Fig. 4). As observed in SEM, the raw poplar had a rigid and compact surface (Fig. 4a). After HCl-BDO pretreatment at 10 mM, large sediment was formed on the fiber surface of pretreated biomass (Fig. 4b), which was probably lignin coalesced/condensed-like structures [9] or “pseudo-lignin” [35]. The sediment had potential to restrict cellulase enzymes to access the fiber surface or bind enzymes irreversibly, thereby having a negative impact on hydrolysis. With higher acid loading like 40 mM, the HCl-BDO pretreated biomass possessed a relatively smooth and clean surface (Fig. 4c), which was in accordance with XPS results that higher acid loading was beneficial to decline lignin coverage on fiber surface (Table 3). Similar trend was also observed in NaOH-BDO pretreatment (Fig. 4d, e). It was proposed that, less lignin coverage was favorable to expose the fiber surface for enzyme attack and decrease the physical barriers of lignin, positively affecting the ease of glucan hydrolysis (Fig. 2h).

High-resolution XPS provided information about types of bonds present at fiber surface (Table 3). The carbon (C1s) area contained component subpeaks around 284.7 eV (C1: C−C, C−H or C=C), 286.6 eV (C2: C−OH or C−O−C) and 288.4 eV (C3: O−C−O or C=O). As listed, the percentage of C1 subpeak decreased with higher acid or alkali loading, while the percentage of C2 subpeak increased. This was probably because 1,4-butanediol (BDO) pretreatment suppressed lignin repolymerization (C–C) and preserved more β-O-4 (C–O–C) linkages of lignin in pretreated biomass [13]. Meanwhile, it was also revealed that alcohols could react to Cα position of lignin to form α-etherified lignin during organosolv pretreatment. As 1,4-BDO contains two hydroxyl groups, the unreacted hydroxyl group introduced a hydroxyl tail at the α position of lignin [13], which increased the percentage of C2 subpeak (C−OH) and promoted lignin’s hydrophilicity. Both suppression of lignin repolymerization [9] and increase of lignin hydrophilicity [36] had potential to mitigate lignin inhibition on glucan hydrolysis through reduced unproductive binding of cellulase enzymes to lignin, resulting in improved ease of glucan hydrolysis.

**Fig. 5:**
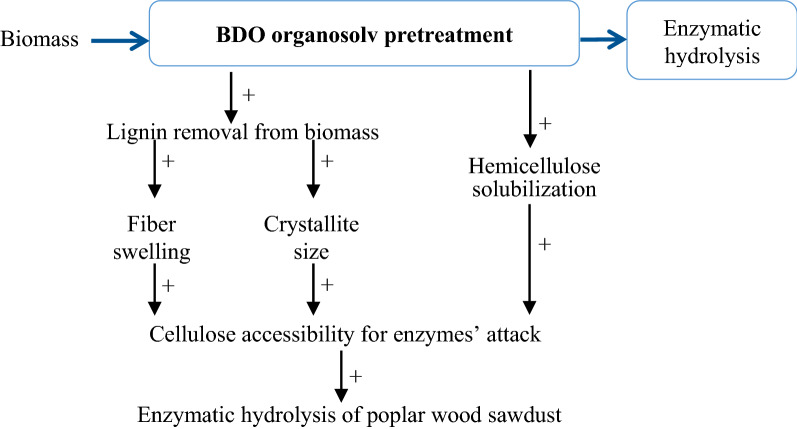
Proposed mechanism of the BDO organosolv pretreatment on improving biomass sacchaarification (+ means improving substrate characteristics or enzymatic hydrolysis)

Results indicated that, acid-catalyzed BDO pretreatment was more effective in improving the enzymatic digestibility of poplar than alkali-catalyzed BDO pretreatment, which was accompanied with greater cellulose accessibility (Fig. 2a) resulted from higher degree of delignification (Fig. 2b) and hemicellulose solubilization (Fig. 2c), as well as the more increase in fiber swelling (Fig. 2e) and cellulose microfibril (Fig. 2g). The hypothetical mechanism model is shown in Fig. 5.

### Effect of acid and alkaline BDO pretreatment on lignin properties

The properties of extracted lignin from BDO pretreatment with alkali or acid were analyzed (Table 4). Evidently, lignin yield increased based on higher acid or alkali loading. Additionally, HCl-BDO pretreatment led to higher lignin yield than NaOH-BDO pretreatment, as more lignin was removed from raw biomass (Table 2) and could be recovered through precipitation of WSF after HCl-BDO pretreatment (Table 4). Result indicated acid-catalyzed organosolv pretreatment is a better approach to fractionate lignin components from lignocellulosic biomass.

**Table 4:** Physiochemical properties of recovered lignin from acid- or alkali-catalyzed BDO pretreatment

	Lignin yield (%)	Mw (g/mol)	Mn (g/mol)	PDI (Mw/Mn)	ATR-FTIR spectra			Antioxidant Capacity (RSI)
Pretreatment					S + G condensed (1329 cm^−1^)	Phenolic OH (1367 cm^−1^)	OH (3338 cm^−1^)	
HCl-BDO 10 mM	9.39	3308	1835	1.80	0.87	0.46	0.49	0.49
HCl-BDO 20 mM	27.10	4038	1992	2.03	0.89	0.55	0.83	0.56
HCl-BDO 30 mM	49.34	4557	2113	2.16	0.90	0.57	0.85	0.60
HCl-BDO 40 mM	66.89	4032	1943	2.08	0.89	0.60	0.86	0.69
HCl-BDO 50 mM	69.66	3056	1698	1.80	0.87	0.60	0.88	0.76
NaOH-BDO 250 mM	34.82	3338	1130	2.95	0.83	0.45	0.50	0.57
NaOH-BDO 300 mM	44.90	3432	1209	2.84	0.88	0.46	0.51	0.57
NaOH-BDO 350 mM	49.21	2692	985	2.73	0.86	0.50	0.67	0.59
NaOH-BDO 400 mM	53.84	2434	1001	2.43	0.80	0.49	0.66	0.60
NaOH-BDO 450 mM	57.22	2039	933	2.19	0.80	0.48	0.55	0.62

As reported, the predominant reactions of lignin during thermochemical pretreatment were depolymerization and repolymerization [37]. The molecular weight distribution analysis could reflect variations of the two reactions of lignin occurring during BDO pretreatments (Table 4). As shown, the molecular weight of HCl-BDO lignin was higher than that of NaOH-BDO lignin, suggesting severer lignin repolymerization reactions during acid pretreatment [33]. Besides, the lignin obtained from HCl-BDO pretreatment at 30 mM had maximum molecular weight, which could be explained by a fact that lignin was subject to a greater extent of repolymerization than depolymerization.

ATR-FTIR was carried out on recovered lignin to determine the physiochemical properties (Fig. 3b). Relative absorbance for each band was calculated as the ratio of the band intensity of different groups to that of C–H vibration of the aromatic ring at 1510 cm^−1^ [38]. The band at 1329 cm^−1^ indicated that the condensed G + S lignin structure [39, 40] was increased with acid loading from 10 to 30 mM and maximized at 30 mM (Table 4), which was in line with GPC results (Table 4). It was also shown that, the formation of phenolic OH groups band at 1367 cm^−1^ [39] increased with acid loading (Table 4). In addition, all the lignin samples showed a broad band between 3400 and 3500 cm^−1^ (Fig. 3b, Table 4), which was attributed to the hydroxyl groups in phenolic and aliphatic structures [39]. The band intensity increased with pretreatment acidity, likely because of more formation of phenolic OH through depolymerization reactions of lignin and/or more BDO condensed to lignin structure that increased the aliphatic OH tails of lignin [13].

Lignin had antioxidant potential, which was the capacity to quench free radicals [41]. Thus, lignin could act as a potential antioxidant in food industry, preventing the loss of food flavor, color, and vitamin content [42]. It was suggested that, lignin compounds that had more phenolic OH groups, fewer aliphatic OH groups, as well as a low molecular weight and narrow PDI might have higher antioxidant activity [43]. As shown, among HCl-BDO lignins, the lignin recovered from HCl-BDO pretreatment at 50 mM had the highest radical scavenging capacity (RSI = 0.76) likely because of its highest amount of phenolic OH group (ratio of 0.60), low molecular weight and narrow PDI (1.80, Table 4). Among NaOH-BDO lignins, the lignin recovered from NaOH-BDO pretreatment at 450 mM had the highest radical scavenging capacity likely due to its lowest molecular weight (Table 4). Results indicated that acid BDO pretreatment resulted in the formation of phenolic hydroxyl groups in lignin, probably increasing its radical scavenging capacity, while alkali BDO pretreatment reduced the molecular weight of lignin, leading to increased radical scavenging capacity. Moreover, results indicated the potential application of BDO organosolv lignin as a natural antioxidant [44]. Natural antioxidants are suggested to be highly desirable in food industry, as the use of synthetic antioxidants in the long term may cause potentially toxicological risks to animal and human health [45].

**Fig. 6:**
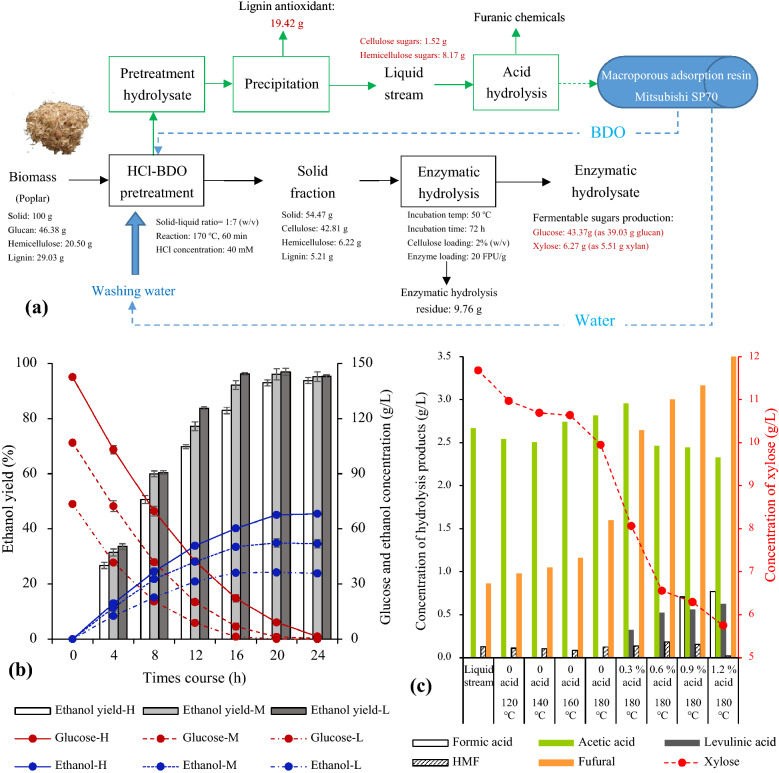
Mass balance of biorefinery based on acid-catalyzed BDO organosolv pretreatment to coproduce fermentable sugars and antioxidant lignin (**a**),  fermentation at different initial glucose concentrations (**b**), concentrations of enzymatic hydrolysis products (c)

Results showed that acid-catalyzed BDO organosolv pretreatment was more effective in improving biomass saccharification (Fig. 1) and producing antioxidant lignin (Table 4) when comparing with alkali-catalyzed BDO pretreatment. Then, mass balance analysis based on acid-catalyzed BDO pretreatment was proposed (Fig. 6). As illustrated, after HCl-BDO pretreatment with HCl loading of 40 mM, the pretreatment hydrolysate containing C5 sugars and lignin was separated from pretreated solid, followed by precipitation. As a result, 19.42 g organosolv lignin was recovered from 100 g raw poplar biomass, which could be used as antioxidant (Table 3). The liquid stream containing 1.52 g cellulose-derived sugars and 8.17 g hemicellulose-derived sugars could be upgraded to furanic platform chemicals [17], like furfural. After acid hydrolysis (1.2 wt% sulphuric acid, 180 °C, 1 h) of the liquid stream, 3.58 g/L furfural could be produced from 11.68 g xylose (detail not shown). BDO solvent was proposed to be well separated from water using Mitsubishi SP70, a kind of macroporous adsorption resin (Mitsubishi Chemical Corporation, Japan) [18]. After separation, BDO could be reused in the pretreatment of lignocellulosic biomass [19], and water could be reused to wash the pretreated solid, reducing the possible inhibition of enzymatic hydrolysis and subsequent fermentation by BDO and degradation products from carbohydrate and lignin. Besides, 39.03 g glucan and 5.51 g xylan in the raw biomass could be converted to fermentable sugars after pretreatment and subsequent enzymatic hydrolysis. The enzymatic hydrolysate was concentrated to different initial glucose concentrations to estimate the fermentability of the enzymatic hydrolysate (Fig. 6). It was shown that 142.51 g/L glucose could be completely consumed at 24 h fermentation. And 68.12 g/L ethanol was produced, with ethanol yield of 93.73%, which verified the fermentability of enzymatic hydrolysate of HCl-BDO pretreated solid. Results suggested that acid-catalyzed BDO pretreatment, which maximized total sugar yield while enabling efficient production of antioxidant lignin from the poplar wood sawdust, had the potential to be a promising pretreatment approach of lignocellulosic biomass, since it not only diversified the bio-based products from biomass, but also encouraged the utilization of lignin as part of sustainable lignocellulosic biorefinery.

## Conclusions

Results showed that BDO organosolv pretreatment was more effective in removing lignin from lignocellulosic biomass as compared to ethanol organosolv pretreatment. Besides, acid-catalyzed BDO pretreatment had better performance in reducing recalcitrance of poplar to achieve reasonable biomass saccharification through enzymatic hydrolysis than alkali-catalyzed BDO pretreatment, which was accompanied with greater cellulose accessibility resulted from higher degree of delignification and hemicellulose solubilization, as well as the more increase in fiber swelling and cellulose microfibril. The maximum sugar yield of 79.41% from original biomass was achieved after HCl-BDO pretreatment (170 °C, 1 h, with 40 mM HCl loading). Moreover, acid BDO pretreatment resulted in the formation of phenolic hydroxyl groups in lignin, which increased radical scavenging capacity of BDO organosolv lignin as a natural antioxidant.

## Materials and methods

### Materials

Poplar sawdust, with a moisture content of 7.33 ± 0.21%, was collected from Xuzhou, Jiangsu Province, China. Chemicals including ethanol, 1,4-butanediol (1,4-BDO, AR, > 99% purity), hydrochloric acid (HCl, 95–98 wt%) and sodium hydroxide (NaOH, AR, > 96% purity) were obtained from Sinopharm Chemical Reagent Co., Ltd. Commercial enzyme blend CTec2 (SAE0020, filter paper activity of 185 FPU/g, protein content of 233 mg/g, endoglucanse, exoglucanase, β-glucosidase, xylanase activity was 2297.8, 114.9, 3451.6 and 8902.9 U/g, respectively) was obtained from Sigma–Aldrich.

### Acid- and alkali-catalyzed BDO pretreatments

Acid-catalyzed ethanol organosolv pretreatment (HCl-ethanol) was performed as follows: 100 g dry mass poplar was soaked in an aqueous solution (ethanol–water ratio of 65:35, v/v) containing 30, 40 and 50 mM HCl at a solid-to-liquid ratio of 1:7 (g: mL). The mixture was pretreated at 170 °C for 60 min.

Acid-catalyzed BDO organosolv pretreatment (HCl-BDO) was performed as follows: 100 g dry mass of poplar was soaked in an aqueous solution (BDO-water ratio of 65:35, v/v) containing 10, 20, 30, 40 and 50 mM HCl at a solid-to-liquid ratio of 1:7 (g: mL). The mixture was pretreated at 170 °C for 60 min [13]. The oil bath (GSC-30L, Yushen Instruments Company, China) was heated to 170 °C during 30 min and maintained at the temperature for 60 min. Alkali-catalyzed BDO pretreatment (NaOH-BDO) was carried out under the same conditions but using an aqueous solution containing 250, 300, 350, 400 and 450 mM NaOH. After pretreatment, the reactor was cooled in tap water. Solid fraction was separated from pretreatment liquor through vacuum filtration, then washed by 2100 mL water. The washed solid, as water-insoluble fraction (WIF), was kept at 4 °C for further use. For HCl-BDO pretreatment, the pretreatment liquor and washing water were combined, as water-soluble fraction (WSF), for lignin recovery by precipitation and determination of the sugar concentration. For NaOH-BDO pretreatment, H_2_SO_4_ was added to pretreatment liquor and washing water to lower the pH to around 2.0 for lignin precipitation. After precipitation, the solid was separated by centrifugation, then rinsed by hot water and freeze-dried for 72 h to recover lignin.

1 $$\text{Lignin yield} (\%)=\frac{\mathrm{Lignin\,recovered\,through\,precipitation }\,(\mathrm{g})}{\mathrm{Lignin\,in\,raw\,biomass }\,(\mathrm{g})}$$

### Enzymatic hydrolysis of the pretreated substrates

Enzymatic hydrolysis was carried out on the BDO pretreated and washed solid at 50 °C, pH 4.8, 180 rpm for 72 h, in air shaker. In enzymatic hydrolysis, cellulose loading was 2% (w/v), and enzyme loading was 20 FPU cellulase/g cellulose. After 72 h enzymatic hydrolysis, flasks were taken out of the air shaker. Samples were taken from enzymatic hydrolysate. Enzymes were inactivated by heating to 100 °C for 5 min and subsequently stored at − 4 °C until sugar analysis was performed by HPLC. All experiments were performed in duplicate.

2 $$\text{Glucan hydrolysis yield} (\%)=\frac{\mathrm{Glucose\,in\,enzymatic\,hydrolysate }\,\left(\mathrm{g}\right)\times 0.9}{\mathrm{Glucan\,loading\,for\,enzymatic\,hydrolysis }\,(\mathrm{g})}\times 100$$

3 $$\text{Xylan hydrolysis yield} (\%)=\frac{\mathrm{Xylose\,in\,enzymatic\,hydrolysate }\,\left(\mathrm{g}\right)\times 0.88}{\mathrm{Xylan\,loading\,for\,enzymatic\,hydrolysis }\,\left(\mathrm{g}\right)} \times 100$$

4 $$\text{Total sugar yield} (\%)=\frac{\mathrm{Sugars\,released\,in\,pretreatment\,and\,enzymatic\,hydrolysis }\,(\mathrm{g})}{\mathrm{Cellulose\,and\,hemicellulose\,in\,raw\,biomass }\,(\mathrm{g})} \times 100$$

### Antioxidant activity of lignin

Antioxidant activity of lignin was measured as radical scavenging activity using 2, 2-diphenyl-1-picrylhydrazyl (DPPH) method. The recovered lignin was dissolved in dioxane/water solution (9/1, v/v), with concentration ranging from 40 mg/L to 200 mg/L. Of the lignin solution, 0.1 mL was added to 3.9 mL of DPPH ethanolic solution (25 mg/L). The mixture was kept at 25 °C for 30 min. Absorbance of the solutions was measured at 517 nm. Inhibition percentage (IP) was calculated [46] and plotted as a function of lignin concentration, in which EC_50_ (lignin concentration needed to obtain 50% IP) was obtained. Radical scavenging index (RSI), as the inverse of EC_50_, was used to evaluate the antioxidant activity of lignin. Higher RSI indicated better antioxidant activity of lignin.

### Fermentation

After enzymatic hydrolysis of HCl (40 Mm)-BDO pretreatment and enzymatic hydrolysis, the enzymatic hydrolysate was concentrated to achieve different initial glucose concentrations of 75 g/L (Low concentration, L), 110 g/L (Medium concentration, M) and 145 g/L (High concentration, H) for fermentation by *Saccharomyces cerevisiae* to produce bioethanol [47]. Glucose fermentation was performed at 30 °C, 100 rpm, pH 5.5 with cell density of OD_600nm_ = 4, for 24 h, The fermentation experiments were done in duplicate.

5 $$\text{Ethanol yield} (\%)=\frac{\text{Weight}\, \text{of}\,\text{produced}\,\text{ethanol}}{0.51\times\text{Weight} \,\text{of}\, \text{glucose}\,\text{in}\, \text{fermentation}} \times 100$$

### Analytical methods

Chemical components of biomass samples were analyzed by following the method developed by the US National Renewable Energy Laboratory [48]. The water-soluble fraction (WSF) was subjected to an acid hydrolysis (4% H_2_SO_4_, 121 °C for 1 h), and the sugars (glucose, xylose, arabinose) in the liquid fraction were determined for mass balance analysis. The sugars and ethanol concentration was determined using a high performance liquid chromatography (HPLC) system (Agilent 1100) with a refractive index (RI) detector. The separation was performed on Bio-rad Aminex HPX-87H column (300 × 7.8 mm) with 5 mM H_2_SO_4_ as the eluent at a flow rate of 0.6 mL/min.

Water retention value (WRV) measurement was performed according to TAPPI UM 256 for evaluation of biomass fiber swelling [49]. To assess cellulose accessibility, staining method by DR28 was carried out as described elsewhere [50]. The crystallinity of biomass samples was measured using an Ultim IV X-ray diffractometer (XRD) equipped with a Cu Kα radiation source (*λ* = 0.15406 nm), which was scanned at the range of 2*θ* = 5° − 50° with a rate of 5°/min. Crystallinity (CrI) was calculated as described before [51]. CrI/cellulose was defined as the ratio of the calculated CrI to the cellulose content of biomass. CrI might represent the total crystallinity in biomass rather than the cellulose itself, and the CrI/cellulose ratio was suggested as an appropriate mean to estimate true crystallinity in native cellulose [52]. The elements and types of bonds present at sample surface were evaluated by high-resolution X-ray photoelectron spectroscopy (XPS) system (Thermo Fisher Scientific, Waltham, US). Surface lignin coverage was calculated according to previous work [49].

Scanning electron microscope (SEM) was used to observe surface morphology of untreated and BDO pretreated samples with different catalyst loadings at magnification of 1 K. The chemical structure of recovered lignin from BDO pretreatment was determined by attenuated total reflection Fourier transform infrared spectra (ATR-FTIR, Spectrum Two, PerkinElmer, US). Spectra of each biomass sample ranged from 500 to 4000 cm^−1^ at a spectral resolution of 4 cm^−1^ with an average from 64 scans. Gel permeation chromatography (GPC, Waters 1525 system, US) equipped with Agilent PL-gel MIXED-C column and Waters 2414 refractive index (RI) detector was used to determine weight-average (Mw) and number-average (Mn) molecular weights of recovered lignin. Polydispersity index (PDI) was calculated as Mw/Mn. Tetrahydrofuran (THF) was used as the mobile phase at a flow rate of 1.0 mL/min. Polystyrene narrow standards were used as calibration standards [53].

## Data Availability

All data generated or analyzed during this study are included in this published article and its Additional file.

## Abbreviations

BDO: 1,4-Butanediol
DPPH: 2, 2-Diphenyl-1-picrylhydrazyl
IP: Inhibition percentage
RSI: Radical scavenging index
WIF: Water-insoluble fraction
WSF: Water-soluble fraction
WRV: Water retention value
XRD: X-ray diffractometer
XPS: X-ray photoelectron spectroscopy
SEM: Scanning electron microscope
ATR-FTIR: Attenuated total reflection Fourier transform infrared spectra
PDI: Polydispersity index
